# International Conference on Population and Development (ICPD) Programme of Action and climate action: an intersecting agenda

**DOI:** 10.1080/26410397.2025.2468574

**Published:** 2025-03-31

**Authors:** Angela Baschieri, Chiagozie Udeh

**Affiliations:** a Science Leader on Climate Health Impacts, The Institute of Environmental Science and Research, Auckland, New Zealand.; b Programme Specialist, Climate Adaptation and Innovation, United Nations Population Fund, Johannesburg, South Africa

**Keywords:** sexual reproductive health and rights, climate change, population and development, gender, youth

## Abstract

2024 marked the 30th anniversary of the International Conference on Population and Development (ICPD) Programme of Action (PoA) held in Cairo in 1994, which coincided with the year that the United Nations Framework Convention on Climate Change (UNFCCC) went into force. Three decades later, these agendas have become increasingly interconnected and climate change has evolved into a climate crisis, as the world undergoes unprecedented demographic changes. How we deal with these unprecedented changes, and the timings of our shared actions, will define our future. This commentary reflects on the principles of the ICPD PoA and argues for their relevance in today’s fight for climate justice. To build a just and sustainable world, climate action must be guided by the aspirations of the ICPD PoA, promoting people-centred solutions, protecting human rights, advancing social justice, ensuring the right to health – including sexual and reproductive health and rights for all – and empowering women and youth in climate action and securing a future in which rights and choices are preserved for every individual.

## Résumé

L’année 2024 a marqué le 30^e^ anniversaire du Programme d’action de la Conférence internationale sur la population et le développement (CIPD), tenue au Caire en 1994, au moment de l’entrée en vigueur de la Convention-cadre des Nations Unies sur les changements climatiques. Trois décennies plus tard, ces programmes sont devenus de plus en plus interconnectés et le changement climatique s’est transformé en crise climatique, alors que le monde connaît des modifications démographiques sans précédent. La manière dont nous gérons ces changements inédits et le calendrier de nos actions communes définira notre avenir. Ce commentaire revient sur les principes du Programme d’action de la CIPD et fait valoir leur pertinence dans la lutte actuelle pour la justice climatique. Afin de construire un monde juste et durable, l’action climatique doit être guidée par les aspirations du Programme d’action de la CIPD et promouvoir des solutions centrées sur les personnes, protéger les droits de l’homme, faire progresser la justice sociale, garantir le droit à la santé, notamment la santé et les droits sexuels et reproductifs pour tous, autonomiser les femmes et les jeunes dans l’action climatique, et garantir un avenir dans lequel les droits et les choix sont préservés pour chaque individu.

## Resumen

En 2024 se celebró el 30^o^ aniversario del Programa de Acción (PdA) de la Conferencia Internacional sobre la Población y el Desarrollo (CIPD), celebrada en El Cairo en 1994, que coincidió con el año en que la Convención Marco de las Naciones Unidas sobre el Cambio Climático (CMNUCC) entró en vigor. Tres décadas después, estas agendas se han vuelto cada vez más interconectadas y el cambio climático ha evolucionado hacia una crisis climática, a medida que el mundo sufre cambios demográficos sin precedentes. La manera en que lidiamos con estos cambios sin precedentes, y los momentos en que tomamos acciones, definirán nuestro futuro. Este comentario refleja sobre los principios del PdA de la CIPD y argumenta a favor de su relevancia en la lucha actual por la justicia climática. Para construir un mundo justo y sostenible, la acción climática debe ser guiada por las aspiraciones del PdA de la CIPD: promover soluciones centradas en las personas, proteger los derechos humanos, fomentar la justicia social, garantizar el derecho a la salud -que incluye SDSR para todas las personas- y empoderar a las mujeres y jóvenes para tomar acción climática y asegurar un futuro donde se preserven los derechos y las opciones de cada persona.

2024 marked the 30th anniversary of the International Conference on Population and Development (ICPD), held in Cairo in 1994, which coincided with the year that the United Nations Framework Convention on Climate Change (UNFCCC) went into force. Three decades later, these agendas have become increasingly interconnected and climate change has evolved into a climate crisis, as the world undergoes unprecedented demographic changes. This commentary reflects on the principles of the ICPD Programme of Action (PoA) and argues for their relevance in today’s fight for climate justice.

## The ICPD PoA and climate action

The ICPD PoA, endorsed by 179 heads of state in 1994, was a landmark agreement to establish a new sustainable development strategy. It recognised that human rights are the building blocks of sustainable development and that we need to invest in gender equality, human capacity at all ages, women's empowerment, and expanded sexual and reproductive health and rights (SRHR) if we are to truly unleash human potential. The ICPD PoA also set a new agenda for sustainability, acknowledging the inevitable linkages between population, development, and the environment, and the need to change the patterns of production and consumption through a unified approach. The PoA also reflected on the rights of migrants, the rights of future generations, and the need to protect them.^1^

In the same year, three conventions came into force as a follow-up to the 1992 Rio Earth Summit. One convention was created to address concerns about biodiversity loss: the UN Convention on Biological Diversity. The second was born of the need to protect land from desertification: the UN Convention to Combat Desertification (UNCCD). The third, UNFCCC, aimed to stabilise greenhouse gas emissions “at a level that would prevent dangerous anthropogenic (human-induced) interference with the climate system”.^2^

In total, 198 countries (called Parties) ratified the UNFCCC and put responsibility on industrialised nations to support the transition to a lower emission process through financial assistance and technological transfer. To underscore the importance of the latter, as part of the Sustainable Development Goals (SDGs), SDG13 calls for all countries to “*take urgent action to combat climate change and its impacts*”*.*^3^ For SDG13, the UNFCCC is the primary intergovernmental forum for coordinating a global response to climate change, and includes targets for developed countries to fund developing countries to limit greenhouse gas emissions, integrate climate change into policies, and plan and support the strengthening of adaptation capacity.^4^

As we celebrate reaching 8 billion people on this planet, it has become clear that the poorest countries, with the lowest per capita (and absolute) emissions and some of the largest populations of young people, face some of the worst impacts of climate change.^5^ Since 1994, the global temperature has been rising steadily, with the average global temperature in 2022 being 1.15°C higher than it was between 1850 and 1900.^6^ According to a recent World Meteorological Organization assessment,^7^ global temperatures will reach unprecedented levels in the next five years due to the warming induced by greenhouse gases and naturally occurring phenomena like El Niño. This study estimates a 98% likelihood that the next five-year period will be the warmest on record.^7^ These rising temperatures and changes in climatic conditions directly and indirectly impact health and the people-centred agenda of sustainable development set by the ICPD PoA, with dramatic consequences for human development, inequality, and our collective prospects to achieve a sustainable and vibrant future. The economic, social, and environmental costs of not prioritising people-centred development in the face of climate crisis are staggering. According to the latest IPCC report on Working Group II on Adaptation and Vulnerability, more than 3.3 billion people currently live in areas that are highly vulnerable to climate change. While vulnerability varies by local geography and social disparities,^8^ it is already leading to widespread damage and loss.^8^

The year 2024 marks the 30th anniversary of the ICPD PoA, and many of the principles underlying the PoA are still relevant in today’s fight for climate justice, underscoring their relevance in developing solutions to “our common agenda”. To build a just and sustainable world, climate action must be shaped by the aspirations of the ICPD PoA, promoting solutions that are people-centred, protecting human rights and advancing social justice, ensuring the right to health including SRHR for all, and empowering women and youth in climate action.

### Promoting people-centred solutions, protecting human rights, and advancing social justice

*The ICPD PoA affirmed that all human beings are born free and equal in terms of dignity and rights. Everyone is entitled to all the rights and freedoms outlined in the Universal Declaration of Human Rights, and is entitled to a healthy and productive life in harmony with nature.* (Principles 1 and 2)

Although human rights were not initially identified as a critical concern in the original formulation of the UNFCCC, the preamble of the Paris Agreement clearly recognised the importance of respecting, promoting, and considering obligations on human rights in taking action on climate change. The Agreement highlights that climate action must protect the rights of the most vulnerable and guarantee the right to development.^9^

*In addition, the ICPD PoA highlights the importance of protecting the rights of indigenous people. We need to ensure that we protect their identity, culture, interests, and support their full participation in their economic, political, and social lives.* (Principle 14)

Indigenous peoples are particularly affected by climate change because of their dependence on and relationship with the environment and its resources.^10^

In 2015, at the 21st Conference of the Parties in Paris, the Local Communities and Indigenous Peoples Platform (LCIPP) was established to increase the recognition of indigenous peoples and local communities and to facilitate their involvement and contribution to both mitigation and adaptation actions, capitalising on their knowledge and insight on nature-based solutions to climate change, in addition to the dedicated space for engagement in intergovernmental climate processes provided by the Indigenous Peoples Organisations (IPO) which is one of the nine observer constituencies of the UNFCCC.^11^

*ICPD Programme of Action underscores the need to provide proper treatment and adequate social welfare services to migrants and their families to ensure that the fundamental human rights of all migrants are protected*. (Principle 12)

As climate change intensifies, it drives the mass migration and displacement of populations affected by rising sea levels, extreme weather events, and ecological degradation. These forced displacements jeopardise human rights, including access to education, health services, and economic opportunities.^12^ A World Bank assessment found that by 2050, without concrete climate and development action, climate change could lead more than 219 million people in 6 regions to migrate within their own countries (86 million in SS Africa, 17 million in Latin America, 40 million in South Asia, 49 million in East Asia and the Pacific, 5 million in Eastern Europe, and 19 million in North Africa). However, the analysis found that if we were to take positive actions to curb global emissions and implement other policies to address the socioeconomic drivers of migration, internal migration could be reduced by 80%.^13,14^

Other populations particularly affected are those living in Small Island Developing States (SIDS). These populations are particularly affected both directly and indirectly through sea level rise and the increased occurrence of cyclones and floods, which affect health infrastructure and economic systems. Changes in both extreme- and slow-onset events increasingly affect low-lying coastal populations, threatening small island habitability. A large portion of the SIDS population will either choose or be forced to migrate or resettle in another area because of climate change.^15^

The climate crisis significantly increases humanitarian needs while hindering effective response efforts, particularly in fragile and conflict-affected regions.^16^ Climate-related migration can disrupt access to health services and education, leading to increased vulnerability to diseases, decreased educational attainment, and long-term socioeconomic disadvantages for displaced populations.^17–19^ As both vulnerable populations and potential host countries grapple with climate pressures, the ICPD PoA’s focus on human dignity and well-being should underpin the required global policy work. More immediately and directly, urgent climate action is needed to address the root causes of climate migration and uphold the rights and opportunities of affected communities and their chances to preserve their homes, communities, and livelihoods.

The lack of a clear legal definition for climate migrants or refugees significantly hinders the development of policies and practices to protect the lives of those affected, limiting their access to rights and support systems in the face of climate-related displacement.^20^ As climate change is expected to drive an increase in global migration, countries should develop more compassionate and inclusive regional and international policies to recognise the legal status and protect the human rights of climate-related migrants. This will include providing comprehensive support and services, including in the area of sexual and reproductive health, to facilitate their full integration into host communities. These challenges underscore the urgency of addressing the humanitarian-development nexus, especially when considering the plight of climate migrants.

*The ICPD Programme of Action emphasizes that development is a fundamental human right that must benefit both current and future generations (principle 3), while recognizing that countries bear different responsibilities based on their economic and historical contexts*. (Principle 15).

These concepts are echoed in climate action frameworks such as the UNFCCC, where wealthier nations, responsible for the majority of global emissions, are urged to lead in reducing carbon footprints and providing financial and technological support to developing countries. However, the stark disparity in emissions – where the wealthiest 10% account for nearly half – reveals the unequal burden of climate impacts, which disproportionately affect the poorest populations.^21^ Moving forward, cities, responsible for 75% of global emissions,^22^ play a pivotal role in shaping sustainable futures.^23^ In 2015 the Paris Agreement,^11^ a legally binding international treaty on climate change adopted in 2015 to keep global emissions under 20°C above pre-industrial levels and further limit temperature rise to 1.50°C by this century, emphasised that “developed country Parties should continue taking the lead by undertaking economy-wide absolute emission reduction targets” and that they meet their responsibility and commitment to a collective goal of mobilising USD 100 billion per year by 2020 for climate action in developing countries.^24^ The future trajectory of our common agenda will depend on how developed countries will fulfil these commitments through adequate climate financing, technology exchanges, and other resources to support adaptation, resilience and recovery from loss and damage, especially to SIDS, the Global South and other vulnerable and affected regions.

All vulnerable groups have the right to be protected from the adverse impacts of climate change, including those who are most at risk, such as women, children, the elderly, Indigenous peoples, people with alternative Sexual Orientation, Gender Identity and Expression, and Sex Characteristics (SOGIESC), low-income communities, migrants, child-headed households, orphans, and people with disabilities. Ensuring their human rights involves integrating their needs into climate policies, promoting social justice, and safeguarding their access to essential services and resources before, during, and after climate-related events. Climate adaptation policies should apply gender equality and social justice principles to ensure that all communities, especially those most vulnerable to the climate crisis and least equipped to respond, have the agency and resources to engage in climate action, including in protecting their SRHR.

### Advancing the right to health for all including SRHR

*According to the ICPD PoA, all individuals have the right to enjoy the highest standard of physical and mental health as well as universal access to healthcare services, including SRHR services*. (Principle 8)

The World Health Organization (WHO) estimates that climate change will cause approximately 5 million excess deaths between 2030 and 2050.^25^ These impacts are experienced directly through the consequences of heat, drought, and extreme weather on health outcomes, and indirectly through anticipated changes in the quality of water, air, and food, as well as through the limitations of the health system in responding to changing patterns of ill-health and maintaining the continuity of services and supplies during emergencies.^26,27^

In the last couple of years, at the UNFCCC COP, more recognition has been given to the health impacts of climate change, with health commitments being agreed at COP26 and with the first Health Day at COP28. Although these developments are delayed, they signal a recognition of the mounting evidence that climate change has a particular impact on health and the need to integrate a health system response to climate action. Climate adaptation responses should aim to be gender-responsive, addressing the gaps identified in various national processes and interventions. Ultimately, the goal is to achieve gender-transformative outcomes by drawing on good practices and documented guidance.^28,29^ The UAE Presidency of COP28 and the WHO, with the support of a number of “country champions”, have developed the COP28 UAE Declaration on climate and health to call for an increase in the proportion of climate financing devoted to health to promote greening of the health system and foster more cross-sectoral collaboration on climate and health. At COP29 the Baku COP Continuity Coalition for Climate and Health was launched, bringing together the presidencies of the host countries of COP26 through COP30 – namely the United Kingdom, Egypt, the United Arab Emirates, Azerbaijan, and Brazil – alongside the WHO with the aim to institutionalise health considerations within the global climate agenda, ensuring that health remains a central focus in climate policies and actions.

The ICPD also reflects on the importance of reproductive healthcare programmes in providing the broadest range of services without any form of coercion, underscoring that all couples and individuals have the basic right to freely decide on the number and spacing of their children (Principle 8).

Over the last 5 to 10 years, more evidence has emerged on the direct and indirect effects of climate change on people's ability to fully realise their SRHR.^30^ Climate-related hazards, including extreme heat, flooding, and wildfires, have a direct impact on anaemia, eclampsia, low birth weight, preterm birth, and miscarriage.^31^ Other studies have found that “an increase of one degree Celsius in the week before delivery is associated with a 6% greater likelihood of stillbirth”,^32^ emphasising the strong linkages of extreme heat exposure to preterm births.^33–36^ Evidence also shows that rising temperatures affect the patterns of vector-borne diseases such as malaria, with adverse maternal and child health outcomes such as maternal anaemia and low birth weight.^37,38^ Furthermore, it has been found that climate variability affects the timing of menarche,^39^ which has long-term effects on bone disease, cardiovascular disease, mental health, and fertility-related problems.^39^ Studies have found that extreme temperatures, floods, and rainfall shocks influence fertility-related outcomes. Some research reports a decrease in fertility rates due to extreme temperatures, while others associate high temperatures with reduced semen quality.^30^ An increase in temperature and air pollution has also been found to affect endocrine disruptors, contributing to the early onset of menopause,^40^ and extreme heat affecting menopausal symptoms^41^ and women’s cancer risks through air pollution, UV radiation, and environmental toxins.^42^

Climate-related emergencies affect access to health services and life-saving commodity supply chains, including contraceptives, emergency contraception, treatment for the prevention of HIV, and safe abortion services.^43–45^ In addition, macro- and micronutrient deficiencies due to food insecurity and undernutrition in pregnant women affect pregnancy and new-born outcomes.^46^ They can lead to low birth weight, miscarriages, and perinatal mortality. Dehydration during pregnancy, caused by a lack of safe water or drought, can be especially devastating to both the mother and child.^46^ Poor socioeconomic conditions due to the climate crisis can further exacerbate period poverty, such as access to menstrual hygiene education, products and sanitation supplies.^47^

*Despite clear and mounting evidence on the impact of climate change on SRHR outcomes at the global level, few countries have addressed the needs of the SRHR in their national climate policies.*^48–50^

This lack of national-level evidence limits country-level capacity to elaborate on effective and targeted policy responses. To improve preparedness and response systems during climate events, there is a need to scale up investments in climate and SRHR research,^51^ knowledge and adaptation, including assessments of populations at risk and investment to assess the population health impacts of climate change. Efforts should prioritise climate-resilient housing, transport, and related public infrastructure. All countries should develop targeted plans for disaster risk reduction, early warning, and early action, including strategies for adapting and delivering essential sexual and reproductive health services in the face of climate and related natural disasters.

### Women at the heart of climate action

*According to the ICPD PoA, empowering women and eliminating violence against them are critical to achieving sustainable development, including women’s ability to control their fertility (Principle 4). The ICPD PoA also underscores the importance of women’s equal participation in civil, cultural, economic, political, and social life at the national level and the importance of eradicating all forms of discrimination on the grounds of sex.* (Principle 4)

Achieving change requires policies and programmes to improve women's access to economic resources and ensure shared responsibilities to eliminate impediments to their participation in public life.

Evidence indicates that climate change disproportionately affects women and girls.^52^ Both the ICPD PoA, which was advocated for and endorsed by women and the UNFCCC, and the Gender Action Plan, which was created as part of the Lima Work Programme on Gender, sought to advance women's participation and promote a gender-transformative climate policy. They recognise the importance of empowering women to alleviate poverty and vulnerability and strengthen their resilience to climate change.

Additional evidence has emerged regarding the influence of environmental and climatic changes on the prevalence of gender-based violence (GBV) and harmful practices, such as child marriage and female genital mutilation/cutting.^53–57^ A global review of national climate policies submitted in recent years shows that only 13 of the 119 countries reviewed have included recognition of or appropriate responses to ensure the protection of women and girls affected by the climate crisis.^48^ In this context, a human rights-based approach that supports individuals in making reproductive choices that best suit their personal circumstances is core to achieving climate justice and protecting the rights of those affected by climate change.

Research shows that several barriers, including cultural norms, childcare responsibilities, inadequate financial support, and limited access to education, hinder women's climate leadership in rural areas.^58^ Effective climate action requires structural changes and the meaningful inclusion of women and girls in climate policy and decision-making at national and international levels. This is crucial to ensure gender-responsive adaptation and the integration of key sectoral issues into climate policies, financing strategies, nationally determined contributions (NDCs), and national adaptation plans (NAPs). Women have been proven to be effective environmental stewards, comprising the majority of the agricultural workforce in developing countries and producing 80% of food despite limited access to critical resources. Empowering women as climate leaders with equal access to resources is essential to combating food insecurity in rain-fed agricultural communities.^59^ Currently, 66% of green economy transitions between 2015 and 2021 were led by men, indicating women may already be left behind.^60^ Climate action must prioritise gender equality in leadership and participation to ensure sustainable progress across the SDGs.

### Young people are at the forefront of climate action

*The ICPD PoA emphasized the importance of investing in young people with education and appropriate SRHR and underscored the importance of protecting the rights of future generations (Principle 2) and the need to give priority to children, ensuring their rights of standards of living and health.* (Principle 11)

*The Lancet* medical journal surveyed 10,000 people and 1,000 children/participants aged 16–25 years in 10 countries (Australia, Brazil, Finland, France, India, Nigeria, the Philippines, Portugal, the UK, and the US) on their thoughts and feelings about climate change and their governments’ responses to related issues. Respondents across all countries were worried about climate change (59% were very or extremely worried). More than 45% of the respondents said that their feelings about climate change negatively affected their daily life and functioning, and many reported a high number of negative thoughts about climate change (83% think people have failed to take care of the planet). Respondents rated government responses to climate change negatively. Climate anxiety and distress were correlated with perceived inadequate government responses and associated feelings of betrayal.^61^

Young people are experiencing ecoanxiety,^62^ that is, anxiety about climate change, which affects reproductive choice and fertility preferences in some contexts.^62^

Even though the Paris Agreement only mentions children and intergenerational equity in its preambular texts, young people are proving to have the biggest global influence in moving the needle on global climate action, successfully problematising global climate inaction and framing their advocacy from a justice perspective.^63^ Through climate strikes and dedicated youth spaces for meaningful youth engagement in climate negotiations, such as YOUNGO, the youth constituency of UNFCCC, the global community is slowly responding to demands championed by youth activists, successfully penetrating the UNFCCC negotiations.^64^ In addition, youth are increasingly reverting to climate litigation as an advocacy tool, with the total number of climate change court cases up from 884 in 2017 to 2,180 in 2022.^65^ At least 34 cases have been brought by, and on behalf of, children and youths aged under 25 years, relying on “*children and youth’s special vulnerability to climate harm and on the principle of intergenerational equity*”; these cases argue that due to their young age, they will bear the effects of climate change for longer.^65^ For similar reasons, it is paramount to ensure that youths have formal and widespread opportunities to define solutions and design a resilient future they will inhabit.

There is a need to prioritise the integration of youth perspectives into climate governance, actively involving young people in decision-making processes. This includes promoting research to deepen understanding of the climate crisis's impact on youth, particularly regarding their economic opportunities, fertility and family aspirations, and mental health.

### Unlocking climate finance for gender and SRHR: bridging the gaps for sustainable action

Significant climate finance is crucial for progress in climate action, including gender and SRHR. Developed countries missed the $100 billion annual climate finance target set at COP15 in Copenhagen in 2009 and only reached that target at the end of 2022,^66^ with loans dominating at 89%, especially from multilateral development banks.^66^ This loan-heavy approach hinders climate action, particularly in vulnerable regions like Africa, where only $29.5 billion was mobilised in 2020, despite needing $250 billion annually.^66^

Adaptation finance remains underfunded at 28%, while gender-focused climate finance is also minimal. Only 1.5% of climate-related official development assistance targets gender equality, and just 2% of adaptation finance is gender-responsive. Few NDCs and NAPs allocate sufficient resources to gender and SRHR.^66^ A UNFPA and QMUL 2023 review of NDCs shows lack of costed interventions across NDCs on SRHR and gender. A similar analysis of adaptation communications submitted by countries shows only 6% address health and well-being.^67^

The next NDC cycle in 2025 (NDC 3.0) presents a chance to prioritise gender and SRHR with dedicated budgets.

## Conclusion

This paper underscores that the ICPD PoA principles remain as vital today as they were three decades ago in guiding climate action that prioritises both people and the planet. Addressing the climate crisis requires a unified global response that confronts the deep-seated inequalities and injustices experienced by various sections of society impacted by the crisis. Given the disproportionate effects of climate change on women and girls, governments should prioritise SRHR and women’s empowerment as essential components of climate resilience. This includes safeguarding health services during climate-related disruptions, ensuring women’s meaningful participation in climate decision-making, and addressing the heightened risks of GBV in communities affected by loss of livelihoods. There is also a need to recognise the increasing displacement of populations due to climate change and adopt policies that protect the protection and well-being of climate-related migrants. Climate action and intervention must ensure the meaningful engagement of youth in all levels and forms of decision-making, recognising them as critical stakeholders whose innovations can unlock key sustainable and locally-led solutions. Progress will rely heavily on substantial climate finance investments that address these key intersections as climate impacts continue increasing in intensity and frequency. The New Collective Quantified Goal (NCQG) on climate finance, which replaces the $100 billion annual target, fails to meet the developing countries’ $1.3 trillion USD target at COP29. This shortfall underscores the urgent need to double efforts to mobilise climate finance from all sources, including the private sector. Strengthening financing is essential to safeguard the gains recorded on the ICPD PoA and build resilience in the most vulnerable communities.

## Provenance statement

This article was not commissioned and went through external peer review.
